# Impact of influent wastewater quality on nitrogen removal rates in multistage treatment wetlands

**DOI:** 10.1007/s11356-014-3647-4

**Published:** 2014-10-11

**Authors:** Magdalena Gajewska, Krzysztof Jóźwiakowski, Ahmed Ghrabi, Fabio Masi

**Affiliations:** 1 Faculty of Civil and Environmental Engineering, Gdańsk University of Technology, Narutowicza st. 11/12, 80-233 Gdańsk, Poland; 2 Faculty of Production Engineering, University of Life Sciences in Lublin, Akademicka St. 13, 20-950 Lublin, Poland; 3 Research Centre for Water Technologies, BP.273, 8020 Soliman Tunis, Tunisia; 4 IRIDRA, Via Alfonso La Marmora 51, 50121 Florence, Italy

**Keywords:** Wastewater composition influence on nitrogen removal, Multistage treatment wetlands, Nitrogen removal, BOD_5_/N ratio, COD/N ratio, C/N ratio

## Abstract

Nitrogen removal in treatment wetlands is influenced by many factors, and the presence of electron donors (biodegradable organic matter) and electron acceptors (nitrate ions) is the main limiting one; for obtaining these conditions, multistage treatment wetlands (MTWs) are required, where an extensive nitrification can be obtained in the first stages under aerobic conditions leaving then to the following anoxic/anaerobic stages the duty of the denitrification. Most of the biodegradable organic matter is however oxidised in the first stages, and therefore, the inlet to the denitrification beds is usually poor of easily degradable carbon sources. This study is comparing the long-term performances obtained at several MTWs operating in Europe (North and South) and North Africa in order to understand if there is a significant avail in making use of the influent chemical oxygen demand (COD)/N ratio during the design phase for ensuring proper performances in terms of N overall removal. The statistic analysis performed in this study have shown that MTWs are capable to ensure sufficient removal of both organic and nutrients even in unfavourable proportions of macronutrients (C and N). The usual assumptions for conventional biological treatment systems concerning adequate C/N ratios seem to be dubious in case of wastewater treatment in MTWs.

## Introduction

Wastewater can have various compositions and, as a consequence, different biodegradability under treatment processes. This fact has a fundamental meaning during treatment processes especially when effective nitrogen removal is required. Nitrification and denitrification are widely accepted as the main processes responsible for nitrogen compounds removal from wastewater in both conventional and treatment wetland plants (Kadlec and Wallace 2009; Makinia et al. 2009; Makinia 2010). These two subsequent processes are conducted by different microbial species with completely different requirements like electrons acceptors or carbon sources. In consequence, the proportions between macronutrients represented by C/N/P become fundamental for efficient biochemical treatment. Usually, wastewater is rich enough in P compounds and phosphorus is not a limiting factor. Whereas, the availability of easily degradable source of carbon expressed as biochemical oxygen demand (BOD)_5_ in total organic matter content (chemical oxygen demand (COD)) in wastewater becomes important for effective nitrogen removal. Thus, the assessment of wastewater susceptibility for biodegradation is most often based on the ratio of COD/BOD_5_, and when effective nitrogen removal is required, also the BOD_5_/total nitrogen (TN) ratio is considered. According to Miksch and Sikora (2010), when COD/BOD_5_ is below 2 and in the same time BOD_5_/TN is over 4, the wastewater is easily degradable and the efficiency for both organic matter and nitrogen removal is over 90 %. This concept is widely accepted for conventional wastewater treatment processes. While in case of treatment wetland (TW) technology it has been suggested that the level of easily degradable organic matter to nitrogen (measured by total Kjeldahl nitrogen (TKN)) to ensure effective nitrification could be like BOD_5_/TKN <1 (Crites et al. 2006; Kadlec and Wallace 2009). This significant difference in approaches could be the results of more complex processes which occur during treatment in wetland systems. According to Kadlec and Wallace (2009), plenty of processes are involved in nitrogen removal in TWs. They can be divided into the following: physical mechanisms which transfer nitrogen compounds from one point to another without results in molecular transformation and biochemical processes which transform nitrogen from one form to another. In such circumstances, the driving forces for nitrogen removal could be significantly different than in conventional treatment plants. Thus, the identification of dependences between COD/BOD_5_ and BOD_5_/TN (or BOD_5_/TKN) ratios and efficiency for nitrogen removal can provide useful information as (i) rate of biodegradability of the studied wastewater and (ii) denitrification capacity of the reactor based on the quantification of the degradable dissolved organic matter, which is fundamental for effective removal of nitrogen in the conventional nitrification/denitrification process.

The objective of the paper is to evaluate the impact of the raw wastewater quality, expressed by BOD_5_/COD and BOD_5_/TN ratios, on kinetics of carbon and nitrogen removals in multistage treatment wetlands (MTWs).

## Materials and methods

This study has been based on already existing data. The facilities have been chosen for their different operational conditions (i.e. temperature): from north and southeast part of Poland, via Italy to the north part of Tunisia as well as different types of wastewater from domestic effluents and storm water to highly polluted reject waters from digested sludge dewatering in centrifuge (reject water treatment) at an existing activated sludge plant in Poland.

The data have been collected only at MTWs which are composed by at least two beds: horizontal subsurface flow (HSSF) and vertical subsurface flow (VSSF) or/and pond. Such facilities are recognised to be more efficient in nitrogen removal thanks to various oxygen and redox potential conditions (Vymazal 2007; Molle et al. 2008; Langergraber et al. 2011; Saeed and Sun 2012).

The investigations were carried out in seven MTWs in Poland: three for single family (SF TWs), two for the local community (local TWs), one for storm water and one for reject water treatment (RWC MTW). In Italy and Tunisia, in each one of the MTWs for local communities, data were collated (Table 1) (Jóźwiakowski 2012; Masi et al. 2013).

**Table 1:** The operation conditions of the MTWs

Plant, location and type	Flow [m day^−1^] pe	Configuration	Area [m^2^]	Hydraulic load [mm day^−1^]	Organic load [g COD m^−2^ day^−1^]
Janów, Poland, SF TW	0.66; 3	VSSF HSSF	18 30 Σ48	37.0	18.7
Dąbrowica I, Poland, SF TW	0.3; 3	VSSF HSSF	24 24 Σ48	12.0	7.0
Dąbrowica II, Poland, SF TW	0.3; 3	HSSF VSSS	24 24 Σ48	12.0	7.0
Darżlubie, Poland, local TW	56.6; 650	HSSF I Cascade bed HSSF II VSSF SSHF III	1200 400 500 500 1000 Σ3350	47.3 141.2 113.4 113.4 56.7	39.0 39.9 24.9 19.5 11.9
Wikono, Poland, local TW	18.6; 220	HSSF I VSSF HSSF II	1050 312 540 Σ1902	19.5 65.7 38.0	12.9 5.7 2.0
Swelina, Poland, storm water		Pond HSSF	5000 m^3^ 960		
Pilot Wschód, RWC	0.24 (5)	VSSF I VSSF II HSSF	7.5 5.0 3.9 Σ16.4	3.2 4.8 28.5	22.8 9.6 5.9
Dicomano, Italy, local TW	525 3500	HSSF I VSSF HSSF II FWS	2 × 500 8 × 210 2 × 900 1600 Σ6080	520.5 313.0 292.0 328.0	105.0 19.0 7.0 2.0
Chorfech, Tunisia, local TW	17.0; 500	HSSF I VSSF HSSF II Reservoir	200 4 × 212.5 2 × 375 50 m^3^ Σ1800	74.0–108.0	244.0 140.0 20.0

The samples of influent and effluent as well as collected with respect to hydraulic retention time after each stage of treatment in MTWs were collected and analysed for BOD_5,_ COD and TN according to the standard methods (APHA 2005).

Mass removal rate (MRR) was calculated on the basis of the following equation:

$$ \mathrm{M}\mathrm{R}\mathrm{R} = \left[\left({C}_{\mathrm{in}}\cdotp {Q}_{\mathrm{in}}\right)-\left({C}_{\mathrm{out}}\cdotp {Q}_{\mathrm{out}}\right)\right]/A\Big[\mathrm{g}/\left({\mathrm{m}}^2\cdotp \mathrm{d}\right] $$

where

- *A* is the area of MTW [m^2^].
- *Q*_in_ and *Q*_out_ are the average influent and effluent flow rates, respectively [m^3^/d].
- *C*_in_ and *C*_out_ average influent and effluent pollutant concentration, respectively [mg/L].

The results were evaluated using the StatSoft STATISTICA 8.0. The normality of variables was checked using the Shapiro-Wilk test (for small amount of samples) with *p* level = 0.05. ‘Box and whiskers plots’ have been chosen as a graphical interpretation of the statistical analysis. The linear relationship was considered significant when *R* ^2^ > 0.8.

## Results and discussion

### Quality of wastewater

Due to different types of wastewater treated and services of analysed MTWs, they were divided into three categories: single family MTWs (SF TWs—three facilities in Poland), local TWs (two facilities in Poland, one in Italy and one in Tunisia) and the others (other MTWs—storm water TW and RWC TW both in Poland). The results were assessed firstly for categories and finally for all MTWs (Table 2).

**Table 2:** The quality of influent wastewater in analysed MTWs

Plant/parameter			TN [mg N L^−1^]	COD [mg O_2_ L^−1^]	BOD_5_ [mg O_2_ L^−1^]	COD/BOD_5_ [−]	BOD_5_/TN [−]
			mean ± *σ* min÷max	mean ± *σ* min÷max	mean ± *σ* min÷max	mean	mean
SF TWs	Janów		82.5 ± 17.2 37.0÷97.0	509.1 ± 99.7 260.0÷610.0	277.7 ± 74.9 104.5÷389.0	1.8	3.4
	Dąbrowica I		134.0 ± 25.4 109.0÷201.0	408.0 ± 94.2 68.0÷300.0	169.0 ± 65.9 99.0÷338.0	2.4	1.3
	Dąbrowica II		134.0 ± 25.4 109.0÷201.0	408.0 ± 94.2 68.0÷300.0	169.0 ± 65.9 99.0÷338.0	2.4	1.3
Local TWs	Wiklno		130.4 ± 9.2 118.4÷148.0	714.6 ± 110.7 508.8÷932.5	382.1 ± 72.0 280.6÷500.7	1.9	3.4
	Darżlubie		120.8 ± 4.3 114.3÷128.9	843.8 ± 40.7 791.4÷901.5	368.7 ± 16.0 340.2÷390.5	2.4	3.1
	Dicomano		28.3 ± 11.9 54.9÷5.7	159.6 ± 102.3 54.9÷5.7	66.5 ± 54.3 175÷2	2.4	2.3
	Chorfech		70.0 ± 98.4 264.0÷4.0	2876.0 ± 879.9 5052.0÷2150.0	1350.0 ± 1205.0 120.0÷2900	2.1	19.3
Others	Wschód		788.1 ± 170.9 710.4÷1789.2	1022.7 ± 93.5 880.0÷1260.0	378.9 ± 87.0 270.8÷569.0	2.7	0.5
	Storm water TW	Dry	8.6 ± 2.1 5.7÷11.3	60.5 ± 16.4 39.8÷72.4	11.6 ± 4.7 8.7÷17.3	5.2	1.4
		Rain	12.4 ± 4.8 8.9÷17.8	98.3 ± 41.0 87.5÷134.8	15.0 ± 4.3 8.8÷23.8	6.6	1.2
		Malt	26.8 ± 7.1 14.6÷41.2	194.3 ± 38.9 102.7÷304.8	38.0 ± 18.4 24.6÷54.6	5.1	1.4

In all analysed MTWs, the quality of discharged wastewater was unstable in time what was confirmed by both very high standard deviation (*σ* > 20 %) and coefficient of variation with the values from 2.0 up to 80 %. The quality of domestic wastewater varied significantly for both nitrogen and organic compounds. The highest concentrations of TN were observed in wastewater discharged into MTWs working in Poland while the highest concentrations of organic (COD and BOD_5_) were observed in MTW working in Tunisia. In MTW working in Italy, both TN and organic concentrations were the lowest and similar to reported by other authors in Europe (Vymazal 2005; Puigagut et al. 2007; Langergraber et al. 2011). Among the analysed wastewater, only two of domestic type were characterised by COD/BOD_5_ below 2 and can be assumed as easily degradable and good source of electron donors for nitrogen removal in denitrification process. As for the BOD_5_/TN ratio, only wastewater in Tunisian MTW had proper values above 4 according to conventional technologies. In many cases, the BOD_5_/TN ratio were far from recommended value, usually much smaller, showing a very high concentration of nitrogen in comparison to the available easily degradable organic matter, like as in reject water or storm water.

### Removal efficiency

**Fig. 1:**
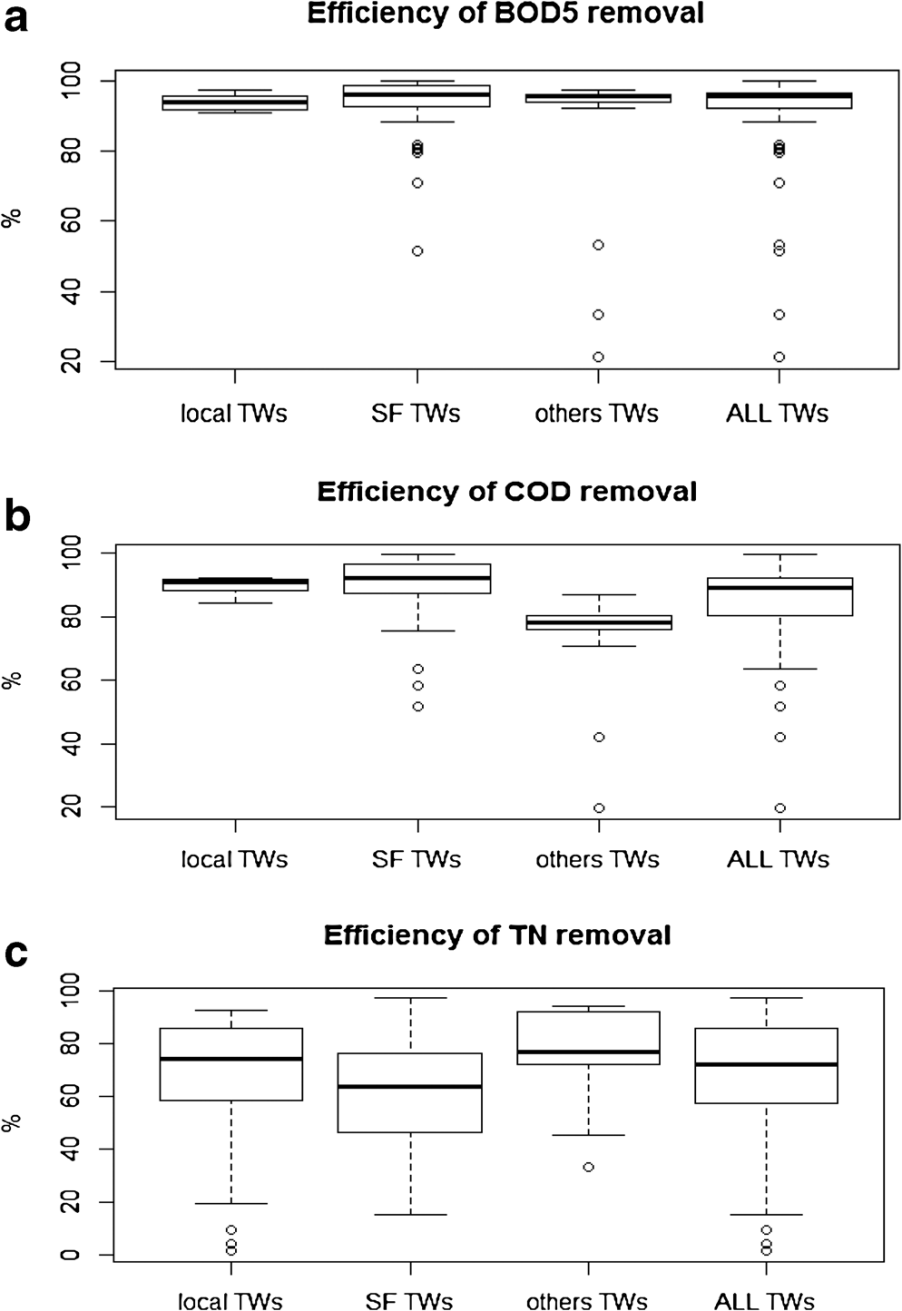
Removal efficiency of organic **a** BOD_5_ and **b** COD as well as **c** TN in analysed TWs

The achieved results on organic matter (OM) removal, especially BOD_5_ (on average over 92 %), confirmed very stable and good performances for all analysed MTWs. A similar behaviour has been observed for COD, with the exceptions of reject water and storm water, with removal efficiency exceeding 85 % (Fig. 1a, b). On the contrary of the OM trends, nitrogen compound removal was unstable and has high variation from 20 to 98 %, shown as whiskers in Fig. 1c. The distribution of the efficiency removal rates represented by box and whiskers plots could indicate that the wastewaters widely considered to be less appropriate for biological treatment (other TWs) when treated in MTWs are providing a more stable TN removal in comparison to MTWs treating domestic wastewater (SF TWs or local TWs).

**Fig. 2:**
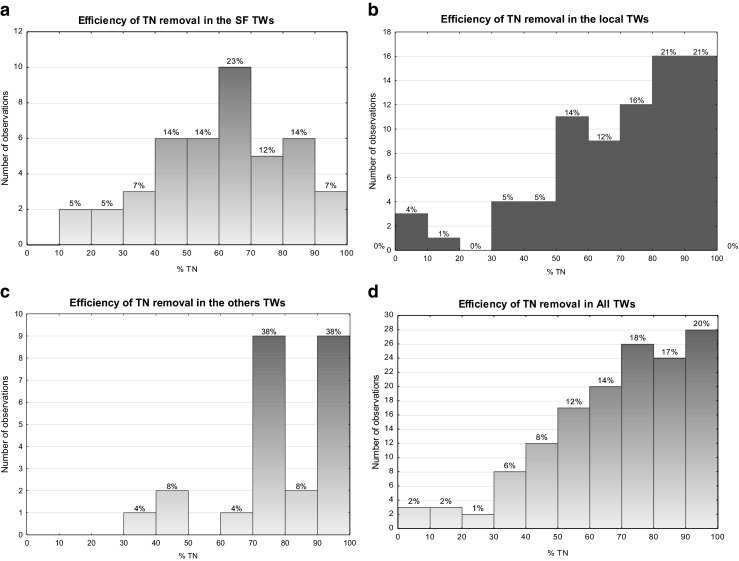
Statistics of TN removal efficiency **a** in SF TWs, **b** in local TWs, **c** in other TWs and **d** in all analysed TWs

Figure 2 delivers information on the occurrence frequency of the nitrogen removal rates for each type of analysed MTWs (a, b, c) and all together (d).

All the monitored MTWs loaded with different types of wastewater secured very efficient removal of nitrogen (Fig. 2d). TN removal performances equal or higher than 50 % occurred in the 81 % of the observations. For domestic wastewater treated in SF TWs, the most frequent removal rates were in the range 60–70 % (Fig. 2a), while for local TWs, TN removal rates over 80 % took place in 33 % of observations (Fig. 2b). In case of other TWs, two ranges of nitrogen removal efficiency removal predominate, 70–80 and 90–100 %: they occurred with the same frequency of 38 % (Fig. 2c). In all cases, nitrogen removal efficiency lower than 30 % has happened exceptionally (Fig. 2a–d).

### Assessment of initial wastewater quality influence on TN efficiency removal

**Fig 3:**
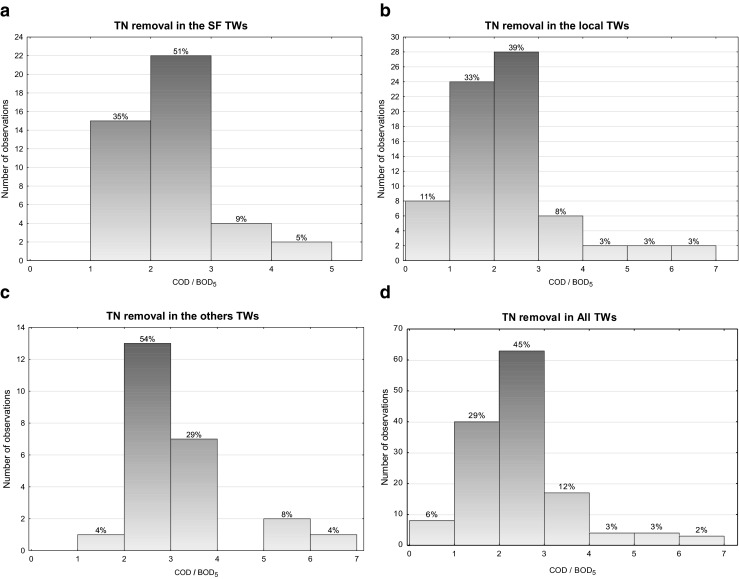
Statistic of COD/1 BOD5 rate frequency

**Fig. 4:**
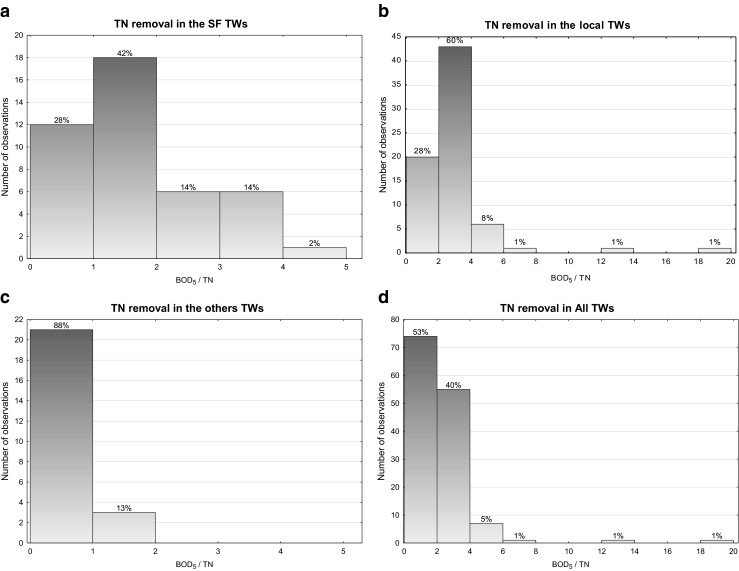
Statistic of BOD_5_/TN rate frequency

Figures 3 and 4 are showing the occurrence frequency of TN efficiency removal for various ratios of COD/BOD_5_ and BOD_5_/TN in analysed cases, respectively. Based on the achieved results, it can be concluded that most frequently, for the analysed types of wastewater, the COD/BOD_5_ ratio was between 2 and 3 and BOD_5_/TN hardly above 4, the recommended value for biological treatments, with the most frequent values in the range 2–4.

**Fig. 5:**
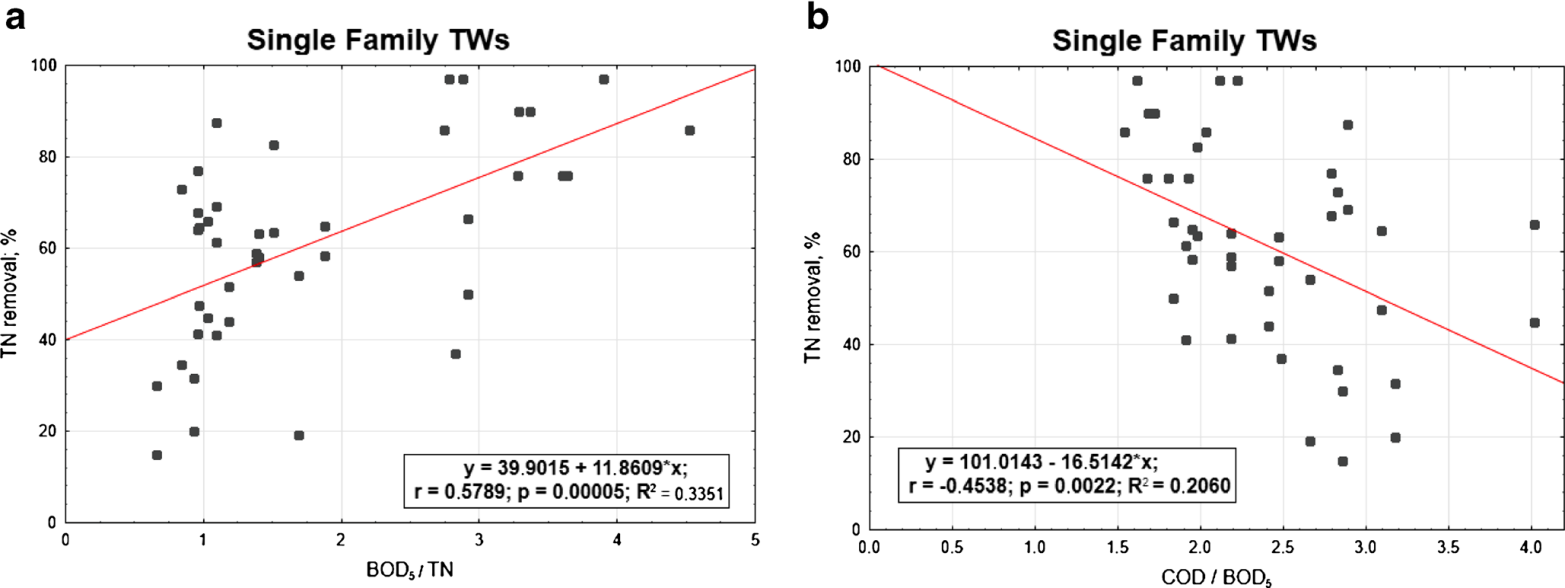
Total nitrogen efficiency vs **a** BOD_5_/TN and **b** COD/BOD_5_ in SF TWs

**Fig. 6:**
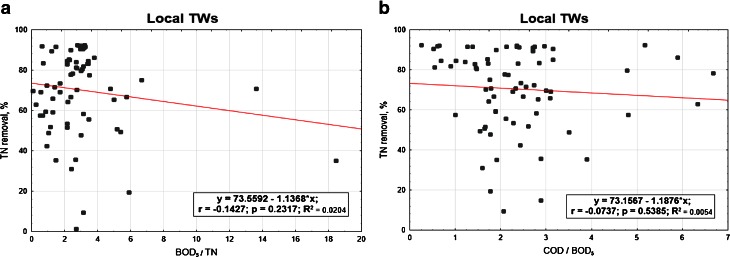
Total nitrogen efficiency vs **a** BOD_5_/TN and **b** COD/BOD_5_ in local TWs

**Fig. 7:**
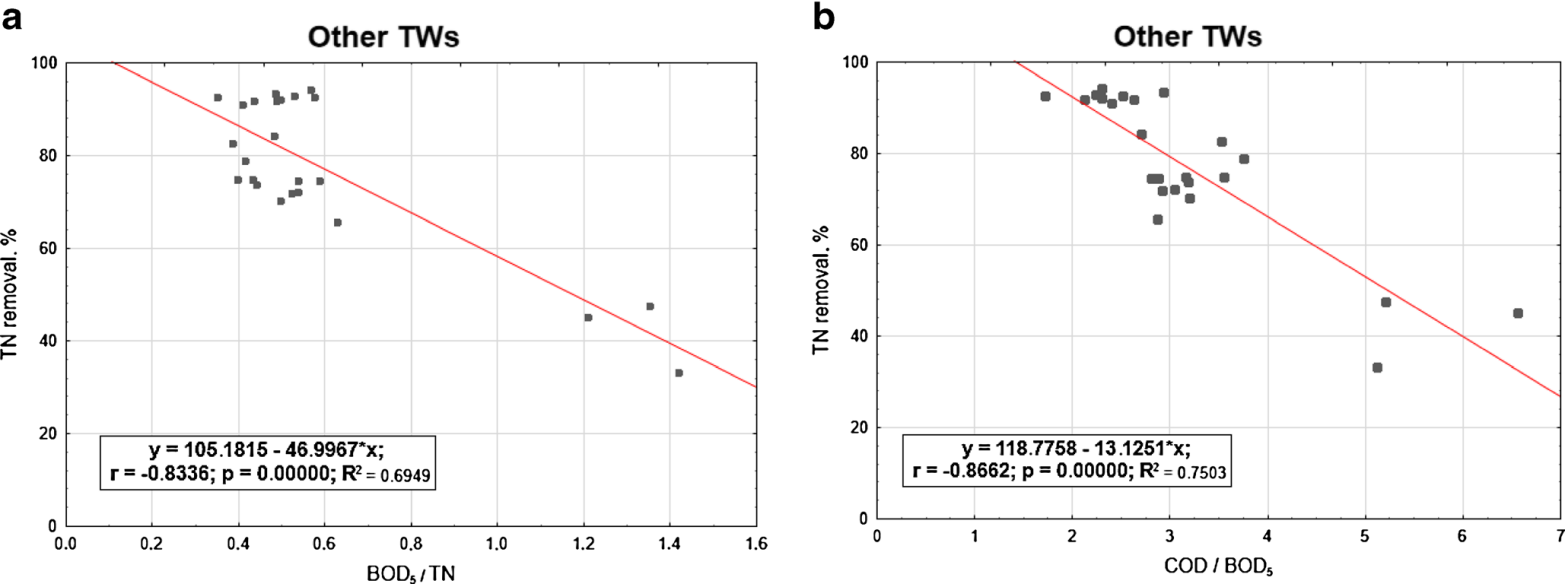
Total nitrogen efficiency vs **a** BOD_5_/TN and **b** COD/BOD_5_ in the other TWs

**Fig. 8:**
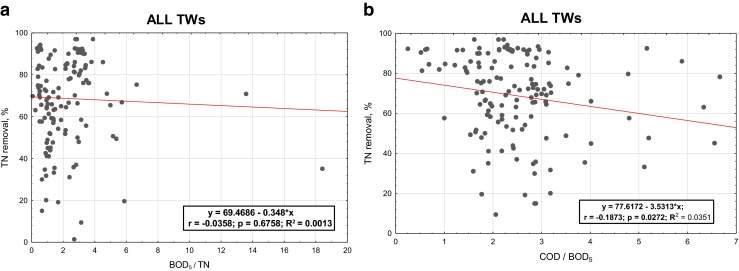
Total nitrogen efficiency vs **a** BOD_5_/TN and **b** COD/BOD_5_ in all analysed TWs

In the next Figs. 5, 6, 7 and 8, the relationships between the nitrogen removal efficiency and ratios between the analysed indicators are presented. The dependency of TN removal efficiency on COD/BOD_5_ ratio is not showing significant differences for all the range of values (Fig. 8b). The most clear but still not significant (*R* ^2^ < 0.8) relation was observed for other TWs, where with the lower value of COD/BOD_5_ the effectiveness of TN is showing a straight increase (Fig. 7b). For the indicator BOD_5_/TN, no significant influence of its value on TN efficiency removal is observed; the highest number of values for the C/N ratio for all the cases are anyway always in the range 1.5–2.5 or even lower as in the case of other TWs (Fig. 7a), where the ratio values corresponding to the highest performances are falling in the range 0.4–0.6; these last results are matching the ones reported by Liu et al. (2013) for their specific case with C/N tending to 0, where also the minimal removal rates are corresponding to the about 30 % found by this study.

The summary of the results of the carried out analysis of statistical significance is presented in Table 3. For the analyses, the *F* test for dependent samples with the significance level *p* = 0.05 was used. Based on the achieved results, it could be assumed that in case of single family TWs and other TWs, the relations are statistically significant (Figs. 5 and 7, Table 3).

**Table 3:** Summary of statistical analyses

Plant type	Regression equations with std err. for coefficient	Factor *x*	*F* value	*p* value of *F* test	*R* ^2^	Adjusted *R* ^2^	*p* value of *t* test for intercept	*p* value of *t* test for *x*
SF TWs	*y* = 39.902 + 11.861 × *x*(5.448) (2.609)	BOD_5_/TN	*F*(1,41) = 20.663	*0.00005*	*0.335*	*0.319*	*0.000000*	*0.000048*
SF TWs	*y* = 101.014 − 16.514 × *x*(12.524) (5.064)	COD/BOD_5_	*F*(1,41) = 10.635	*0.00224*	*0.454*	*0.187*	*0.000000*	*0.002238*
Local TWs	*y* = 73.559 − 1.137 × *x*(3.719) (0.943)	BOD_5_/TN	*F*(1,70) = 1.456	0.23171	0.020	0.006	0.000000	0.231708
Local TWs	*y* = 73.157 − 1.188 × *x*(5.090) (1.921)	COD/BOD_5_	*F*(1,70) = 0.382	0.53846	0.005	–	0.000000	0.538460
Other TWs	*y* = 105.182 − 46.9967 × *x*(4.362) (6.639)	BOD_5_/TN	*F*(1,22) = 50.110	*0.00000*	*0.695*	*0.681*	*0.000000*	*0.000000*
Other TWs	*y* = 118.7758 − 13.1251 × *x*(5.372) (1.614)	COD/BOD_5_	*F*(1,22) = 66.094	*0.00000*	*0.750*	*0.739*	*0.000000*	*0.000000*
All TWs	*y* = 69.469 − 0.348 × *x*(2.558) (0.830)	BOD_5_/TN	*F*(1,137) = 0.176	0.67582	0.001	–	0.000000	0.675825
All TWs	*y* = 77.617 − 3.531 × *x*(4.316) (1.582)	COD/BOD_5_	*F*(1,137) = 4.982	0.02724	0.035	0.028	0.000000	0.027238

Significance of values in italic is *p* level = 0.05

There are several causes that can bring to the still good performances of MTWs with unfavourable wastewater composition and lack of nutrients: basing on this study, where some long-term monitoring sets of data, taken at MTWs located in different locations and very diverse climatic operating conditions, it can be noted that the biological processes involved in nitrogen transformation are proven to be also in MTWs dependent on the specific concentrations of nitrogen forms and organic matter and also in the overall composition and the ratios between them, even though, with a different behaviour if comparing with other biological treatment schemes, like as activated sludge systems, MTWs appear to be creating favourable conditions that can ensure a high TN removal efficiency also with a lack of nutrients in the inlet wastewater, as observed by several researchers in the past years (Tanner et al. 2002; Vymazal 2007; Masi 2008; Kadlec and Wallace 2009; Zhao et al. 2010; Saeed and Sun 2012; Ding et al. 2012; Liu et al. 2013). The most efficient physical processes contributing to nitrogen transformations in MTWs are filtration and adsorption: the organic matter transported by the inlet wastewater, as particulate or colloids, is trapped into the beds and then it is slowly degraded, producing smaller and more easily biodegradable molecules that can act as electron donors for denitrification; a similar behaviour can be related to the presence in the beds of dead tissues of plants or biofilm (Masi 2008; Kadlec and Wallace 2009). Other factors that can explain the scarce dependence in MTWs of TN overall removal on the inlet composition are the so-called unconventional biochemical processes like anaerobic ammonium oxidation (Anammox) or CANON, as it has been proven by the studies of Dong and Sun (2007), Faulwetter et al. (2009) and Gajewska and Ambroch (2012), where it is clearly shown that TWs can achieve good performances in the treatment of wastewater with high ammonia and low organic content, with active Anammox even with wastewater temperature below 20 °C.

## Conclusions

Basing on the carried out analysis of the achieved results of the investigation, the following conclusions can be made:

1. The analysed facilities are confirming that MTWs can provide good removal rates for both organic matter and nitrogen also in high-strength wastewater treatment facilities and with unfavourable inlet composition, expressed by the indicators COD/BOD_5_ and BOD_5_/TN.
2. For the design of MTWs, there is a limited usefulness of the commonly used indicators in conventional treatment technologies, like COD/BOD_5_ and BOD_5_/TN ratios.
3. The statistical analysis performed on all the studied facilities is showing that values of the indicator BOD_5_/TN in the range 1.5–2.5 are the most common for the highest N removal rates obtained in the observed monitoring periods; the values, lower than the usual 4–5 that are commonly observed in other biological treatment methods, can be explained by the unique capacity of wetland systems in providing endogenous sources of biodegradable OM, in trapping the slowly degradable OM with a progressive release of smaller and more bio-reactive molecules and finally in ensuring appropriate environments for the simultaneous combination of alternative nitrogen transformation processes that are not needing the same operating conditions of the nitrification/denitrification pathway.
